# Equivalent Heat Source Model of Thermal Relay Contact Based on Surface Roughness of Silver–Magnesium–Nickel Contact

**DOI:** 10.3390/ma17225583

**Published:** 2024-11-15

**Authors:** Bo Li, Huimin Liang, Pinmou Li, Yuexian Li, Aobo Wang

**Affiliations:** 1 Institute of Reliability in Electrical Apparatus and Electronics, Harbin Institute of Technology, Harbin 150001, China; libohit@hit.edu.cn (B.L.); hitra@hit.edu.cn (H.L.); 2 Harbin Institute of Technology School of Future Technology, Harbin Institute of Technology, Harbin 150001, China; 2021113239@stu.hit.edu.cn (P.L.); 2024111515@stu.hit.edu.cn (Y.L.)

**Keywords:** equivalent heat source, arc, HSER, surface roughness

## Abstract

In a sealed electromagnetic relay, the change in the surface roughness mainly depends on the collision wear between the contact and the moving reed and the ablation effect of the arc on the contact surface based on the strong correlation between the contact resistance and the surface roughness of the Ag-Mg-Ni contact. With a change in contact resistance, the contact temperature increase in a hermetically sealed electromagnetic relay (HSER) is greatly affected. Under extreme overload conditions, the contact surface is severely ablated by the arc, and the roughness increases rapidly with the number of cycles, which greatly affects the contact resistance of the contact surface and the reliability of the relay. A thermal model of a relay contact system based on the surface roughness of Ag-Mg-Ni contacts was established in this paper by analyzing the effect of an arc on the surface roughness of Ag-Mg-Ni contacts under heavy overload conditions. The arc image of the Ag-Mg-Ni contact was recorded using a double-axis arc photographing platform, and the moving track of the arc center under overload conditions was drawn. This paper explored the patterns of arc center movement on the contact surface and the effects of the arc on the surface roughness of the contacts by analyzing the probabilities of the arc center appearing in various locations. A mathematical model correlating the number of contact cycles with contact resistance was established. Subsequently, a finite element simulation model for the equivalent heat source of the contact was developed. The theoretical model error was less than 10%. The accuracy of the equivalent heat source model was verified by comparing the measured data with the simulation results.

## 1. Introduction

As the class of relay with the highest reliability, sealed electromagnetic relays have great airtightness and environmental adaptability when working in a harsh environment.

Under normal working conditions, the main heat sources of a relay include arcing heat during the transient breaking process, heat from steady-state contact resistance, and heat from coil energization. Due to the significant enhancement of microhardness and conductivity, the silver–magnesium–nickel strip alloy after internal oxidation treatment has excellent electrical conductivity, thermal conductivity, and creep resistance; elastic properties at a high temperature; and excellent arc erosion resistance and mechanical wear resistance. Accordingly, the alloy is an indispensable elastic reed and contact material for electromechanical components such as electromagnetic relays, connectors, and switches [1]. With a low breaking time, the effect of the arc on silver–magnesium–nickel contact is slight. However, with the increase in breaking time or load level, the impact gradually increases, and the erosion degree of the arc continues to accumulate, gradually changing the surface morphology and roughness of the silver–magnesium–nickel contact, resulting in great changes in the contact resistance, which continuously degrades the contact system, thus losing effectiveness [2,3,4]. Therefore, the establishment of the equivalent heat source of thermal relay contact based on the contact surface roughness is important to research.

During the operation and release processes of the relay, when the load voltage exceeds 12–20 V and the load current reaches 0.25–1 A, an arc [5,6,7] is generated in the Ag-Mg-Ni contact gap. The arc temperature is extremely high, and the energy contained is also very high. Due to these characteristics of the arc, the Ag-Mg-Ni contact of the relay is severely eroded as the arc is generated, leading to the melting of the surface of the silver–magnesium–nickel contact and a loss of quality. In recent years, numerous scholars have conducted extensive research on arc characteristics [8,9,10,11]. The relationship between arc duration, contact resistance, and electrode mass loss was obtained in a study of arc generation by contactors under 0.5-, 0.75-, 1.0-, 1.25-, and 1.5-times load [12]. Under the conditions of DC 300~1000 V/1000 A, experiments were conducted on the breaking resistance and arc characteristics of inductive loads with different time constants in the contactors’ self-magnetic field arc extinguishing chambers. The variation patterns between the arcing time, breaking voltage, and time constant were obtained [13]. By establishing the MHD arc simulation model of a metal grid, the movement process of the arc in the grid was researched, and the relationship between the arc root generation and the electric field intensity and current density as well as the movement law of the arc was analyzed [14]. The contact surface micro-morphology was photographed, and the influence of arc energy on the contact surface micro-morphology was explored by analyzing the arcing and ablation characteristics of a CuCr55 electrode contact vacuum arc [15].

Generally, the contact resistance of a relay increases with the increase in surface roughness. Surface roughness refers to the micro-geometric characteristics composed of small spacing and peak valleys on a machined surface. A commonly used roughness measurement is the arithmetic mean roughness value R_A_. Other commonly used roughness parameters include R_V_ (maximum contour valley depth), R_Z_ (maximum contour height), and R_Q_ (root mean square deviation of contour) [16,17,18]. The surface roughness model was first developed by Greenwood J. A, and other scholars proposed and established the Greenwood–Williamson contact model. Based on the G-W model, scholars continue to optimize the finite element model to make up for the defects of the roughness model. The surface roughness model is also being optimized [19,20,21,22]. The relationship between the contact resistance and the arithmetic mean Ra of the rough surface profile is derived based on the modified G-W model [23]. The correlation between the surface roughness parameters and the contact resistance of dynamic and static contacts was analyzed using the gray correlation analysis method. The correlation between the surface roughness of dynamic and static contacts and the contact resistance was strong, and the correlation between the surface morphology of dynamic contacts and the contact resistance was stronger [24]. A correlation model between the three-dimensional shape parameters and the contact resistance was established by collecting the three-dimensional shape information and SEM (scanning electron microscope) images of the contact surface and using the gray correlation analysis method to screen the shape parameters. This model was combined with a BP neural network [25].

The working temperature of the relay also rises with the release of a large amount of heat. At a high temperature, the conductivity of relay contact changes greatly, and the deterioration degree of the contact surface is increased, which greatly affects the life of the relay [26]. In recent years, numerous scholars have conducted extensive research on the thermal contacts of relays [27,28,29,30]. The steady-state heat of the magnetic holding relay was analyzed through ANSYS Workbench 17.0 software, and the distribution of Joule heat between the contact surfaces was obtained when the contact surfaces changed with the current level after repeated short-term work [31]. A theoretical analysis method of the contact pressure, loop resistance, and contact temperature rise of the electromagnetic relay contact spring system was proposed, and the mathematical relationship between the push rod stroke and the contact pressure, loop resistance, and the contact temperature rise was established. A mechanical–electrical–thermal direct coupling simulation analysis method of a contact spring system based on COMSOL multiphysics finite element software was built [32].

The research on the influence of relay surface roughness on the contact temperature rise and the establishment of the equivalent heat source model based on roughness are not only of great significance for predicting relay life but also provide an important reference for optimizing relay structure. This is helpful in reducing the contact temperature rise, improving reliability, and prolonging the life of relays. 

**Figure 1:**
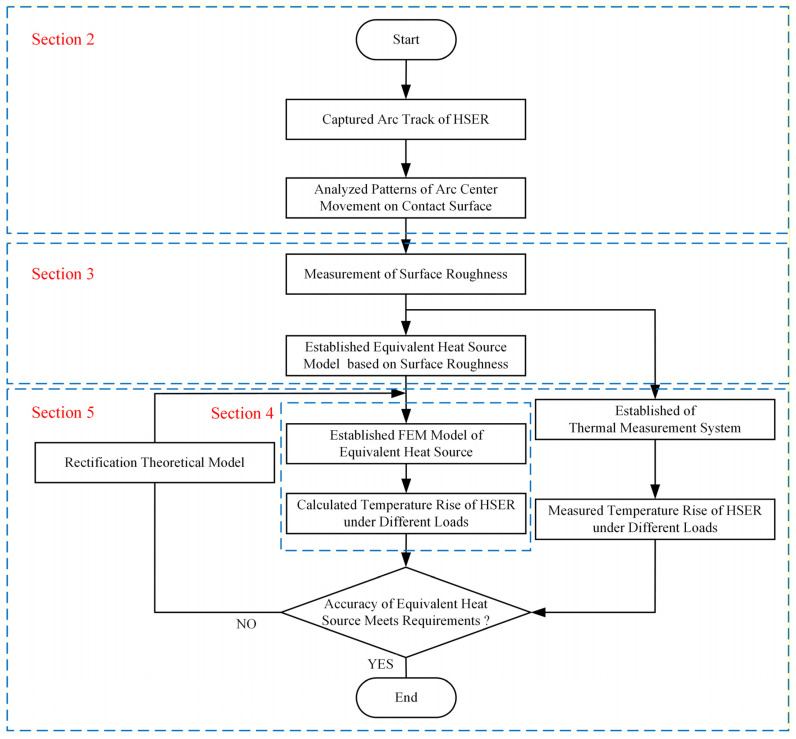
Flowchart of research in this paper.

The effect of the arc on the surface roughness of Ag-Mg-Ni contact was studied in this work, and the thermal model of the relay contact system based on the surface roughness of Ag-Mg-Ni contact was established. The arc images of the Ag-Mg-Ni contact were recorded using an arc camera system, and a moving track of the arc center under overload conditions was drawn. The arc movement law and the influence of the arc on the surface roughness of the contact were studied by calculating the probability of the arc center appearing in different positions. The contact cycles–contact resistance model was established. Then, the finite element simulation model of the equivalent heat source was established, and the measured data were compared with the simulation results. The structure of this paper is shown in Figure 1.

## 2. Influence of Contact Arc of Sealed Electromagnetic Relay on Surface Roughness

### 2.1. Composition and Construction of Double-Axis Arc Photographing Platform

No less than two-dimensional synchronous observation is required to capture the motion trajectory of an arc. Therefore, the double-axis arc photographing platform for the test was mainly composed of two high-speed cameras, a relay reed push and synchronous signal device, two computers, a load box, a sodium lamp, and two power supply groups.

**Figure 2:**
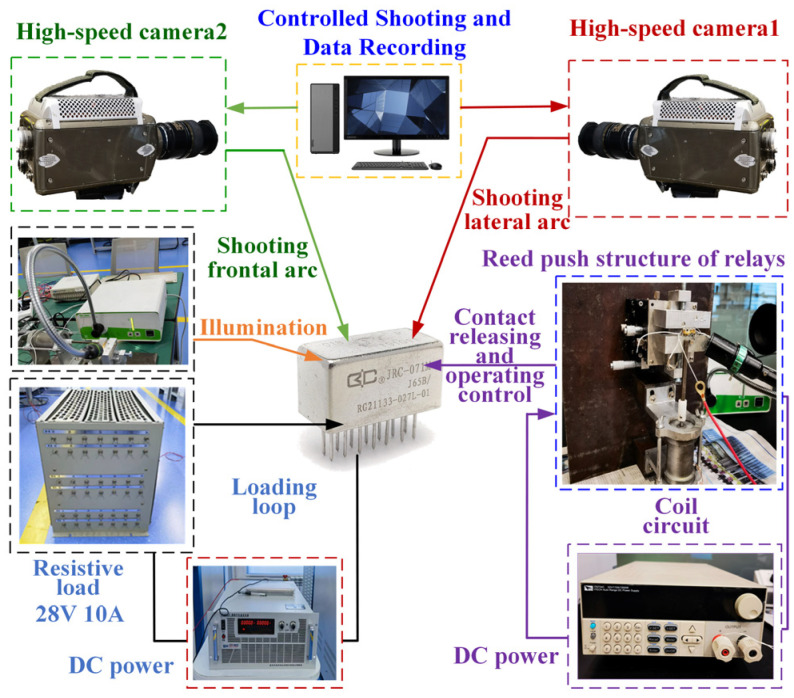
Overload double-axis arc photographing platform.

The relay contact system was fixed on the fixture of the relay reed pushing mechanism, and two high-speed cameras were set up on the front and side dimensions of the contact system to capture the arc tracks on the front and side for later data processing. The frame rate of the high-speed cameras was set to 40,000 FPS. Due to the short exposure time and very dark image, the relay base plate was directly exposed using the white sodium lamp to improve the brightness of the image. The Ag-Mg-Ni contacts of the relay were loaded, and the operation and release between the Ag-Mg-Ni contacts were achieved using another power supply to control the relay reed pushing mechanism. The structure of the double-axis arc photographing platform is shown in Figure 2.

### 2.2. Analysis of Arc Dynamic Process and Drawing of Arc Trajectory

Due to the extremely short arcing time, the arc images of the relay during operation and release states were recorded using high-speed cameras, and the arc images were rendered into an image sequence according to the frame numbers. The position of each arc’s burning center was calculated in proportion to the size of the Ag-Mg-Ni contact.

**Figure 3:**
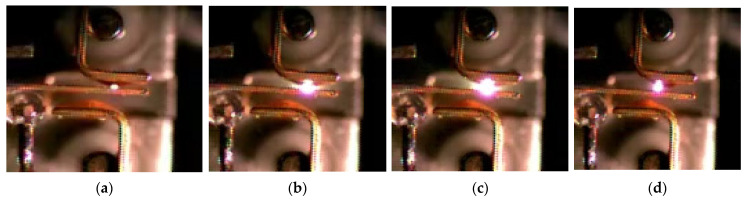
Direct view (side) of arc image in positive Y direction of NO contact: (**a**) is the arc image at frame 0 (arcing point); (**b**) is the arc image at frame 6 (0.15 ms); (**c**) is the arc image at frame 12 (0.3 ms); (**d**) is the arc image at frame 15 (0.38 ms).

**Figure 4:**
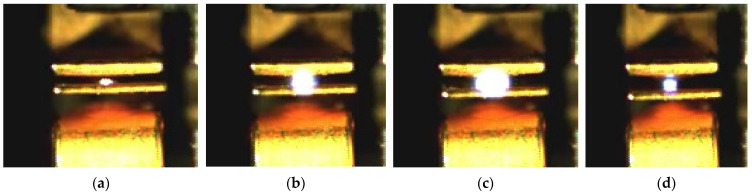
Direct view (front) of arc image in positive X direction of NO contact: (**a**) is the arc image at frame 0 (arcing point); (**b**) is the arc image at frame 6 (0.15 ms); (**c**) is the arc image at frame 12 (0.3 ms); (**d**) is the arc image at frame 15 (0.38 ms).

The arc images of the NO contact of the relay captured by high-speed cameras are shown in Figure 3 and Figure 4.

**Figure 5:**
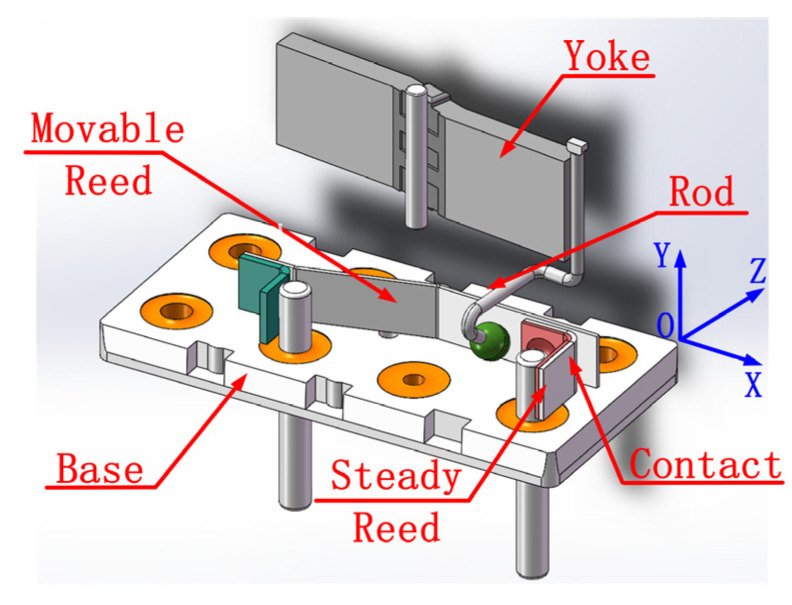
Schematic diagram of coordinate axis direction.

The following coordinate axes were established to determine the specific position of the arc trajectory and draw a two-dimensional arc trajectory diagram: the corner of the contact is the origin, the side length of the front of the contact is the *Y* axis, and the side length of the contact is the *X* axis, as shown in Figure 5.

Taking the coordinate axis as the reference platform, the arc images were processed according to the scale and contact size. The partial data for the arc center position on the surface of the NO contact were obtained as shown in Table 1.

**Figure 6:**
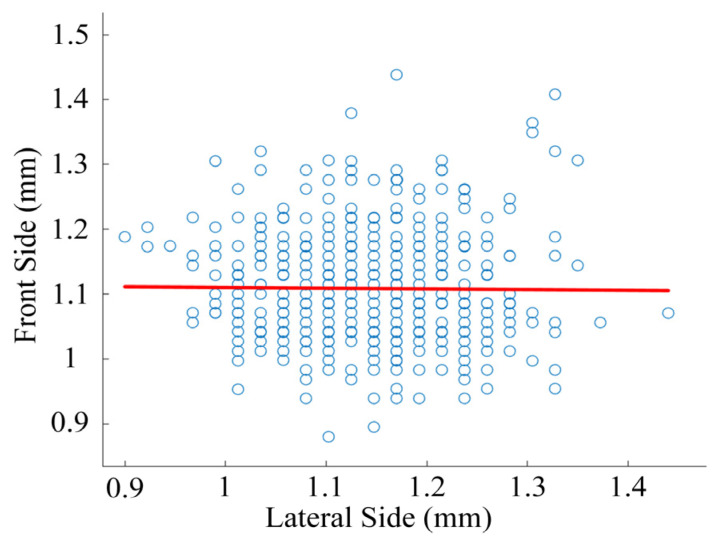
Arc center scatter of NO contact.

Based on the above arc center trajectory data, the arc center scatter distribution diagram was drawn, as shown in Figure 6.

As shown in Figure 6, the center point of the arc is almost concentrated in a certain region on the contact. According to the analyzed arc images, the region is the contact region between the movable reed and the contact convex part. In this region, the distribution law of the arc center is that the arc center is distributed symmetrically along the centerline. The closer to the centerline, the denser the distribution of the arc center and the greater the probability of its occurrence. On the contrary, the farther away from the centerline, the sparser the distribution of the arc center and the smaller the probability of its occurrence. According to the above data, the position of the arc center follows a certain rule: it mainly appears near the contact point between the contact and the movable reed and changes with the contact morphology. As the contact points of the reed and the contact move continuously, they appear near the corresponding new contact points, and the connecting lines of these contact points are regarded as the centerline.

**Figure 7:**
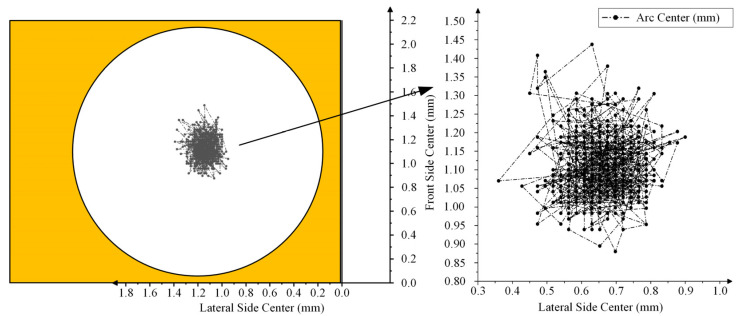
Arc center trajectory of NO contact.

The arc center trajectory of the dynamic contact was drawn based on the position of the dynamic contact arc in the coordinate axis in Table 1, as shown in Figure 7.

**Figure 8:**
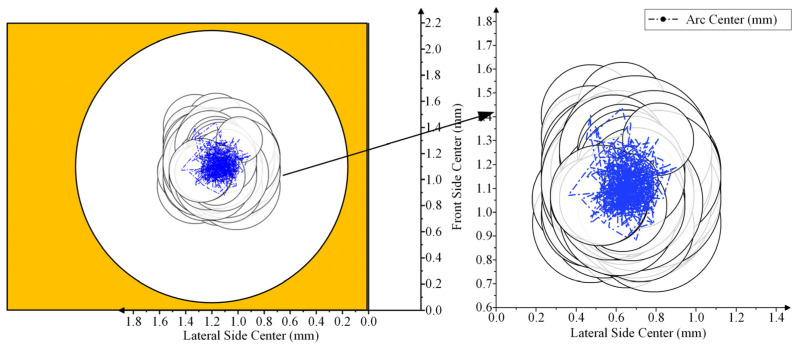
Arc ablation region distribution of NO contact.

Considering the radius of the ablation region of the arc on the contact surface, the region is approximately circular in the plane. The distribution diagram of the arc ablation region on the NO contact was drawn, as shown in Figure 8.

The contact surface morphology was photographed to verify whether the arc center trajectory and arc ablation region distribution map drawn based on the image conform to the actual distribution and to judge the center trajectory error. The specific morphology of the contact surface was photographed under an optical microscope.

**Figure 9:**
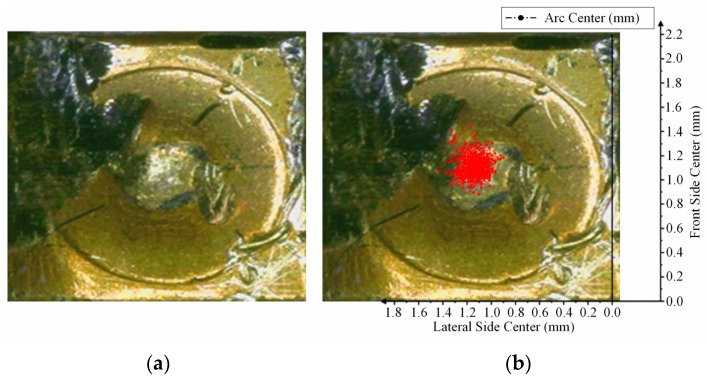
Comparison of arc center trajectories of NO contact: (**a**) is a photograph of the contact morphology; (**b**) is the comparison between the arc center track and contact.

**Figure 10:**
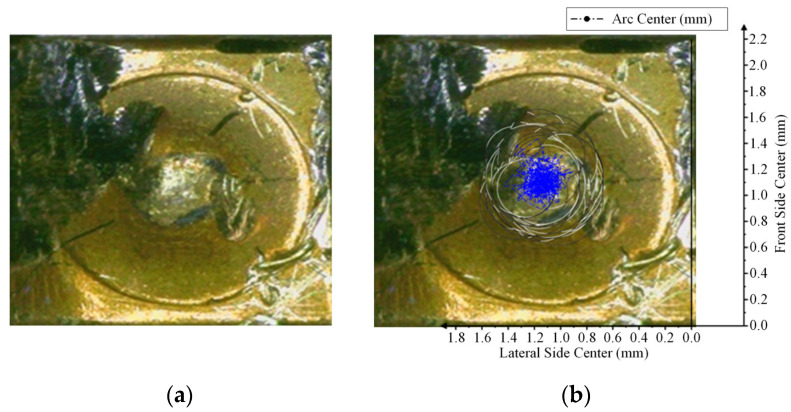
Comparison of arc ablation distribution of NO contact: (**a**) is a photograph of the contact morphology; (**b**) is the comparison between the arc ablation distribution and contact.

A comparison of the arc center trajectory and arc ablation region distribution of the NO contact with the contact morphology is shown in Figure 9 and Figure 10.

Assuming that the arc energy is mainly concentrated in the arc center, the arc center is the main factor causing the center contact ablation. The accuracy of the moving trajectory can be determined by comparing the coincidence of the arc center trajectory and contact ablation.

According to Figure 10, the contact surface ablated by the arc is obviously uneven. The inner material of the most severely ablated region is exposed. Black spots are also visible on the contact surface, which are judged to be the black oxide layer that formed on the contact surface material at a high arc temperature.

By overlapping the arc center trajectory and arc region distribution with the ablated region, the arc center trajectory basically coincides with the most severely ablated region on the contact surface, the error of which is less than 10%. This image is regarded as an approximate objective reflection of the arc center movement trajectory on the contact surface. Most of the black oxidation region on the contact surface is also covered by the distribution map of the arc ablation region.

### 2.3. Influence of Arc on Contact Surface Roughness

According to the above analysis, the arc center is mainly concentrated near the contact point between the contact and the NO reed. The occurrence probability of the arc center in each region was analyzed for different numbers of relay cycles to determine the movement of the arc center trajectory and to describe the movement law of the arc center.

**Figure 11:**
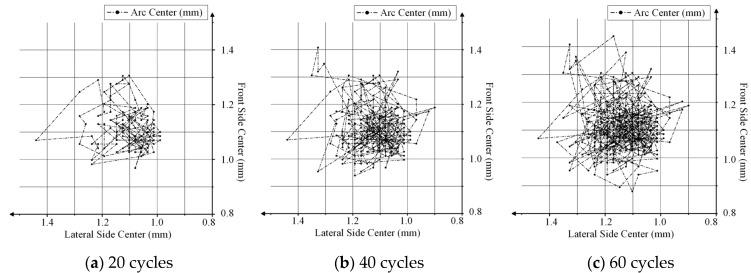

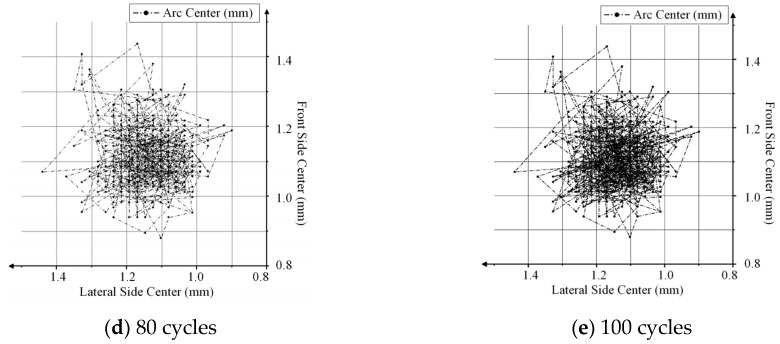
Arc center trajectory distribution for different numbers of cycles of NO contact: (**a**) is the arc center trajectory distribution after 20 cycles; (**b**) is the arc center trajectory distribution after 40 cycles; (**c**) is the arc center trajectory distribution after 60 cycles; (**d**) is the arc center trajectory distribution after 80 cycles; and (**e**) is the arc center trajectory distribution after 100 cycles.

Take the arc center trajectory diagram of the dynamic contact with different numbers of cycles as an example for meshing. The image after meshing is shown in Figure 11.

According to Figure 11, the arc center is entirely concentrated in a certain region in the center of the NO contact. With an increase in the number of cycles, the arc center is obviously more concentrated in a small region with reciprocating movement, occasionally moving outward. The occurrence probabilities of an arc center in the grid region of the NO contact part are shown in Table 2.

**Figure 12:**
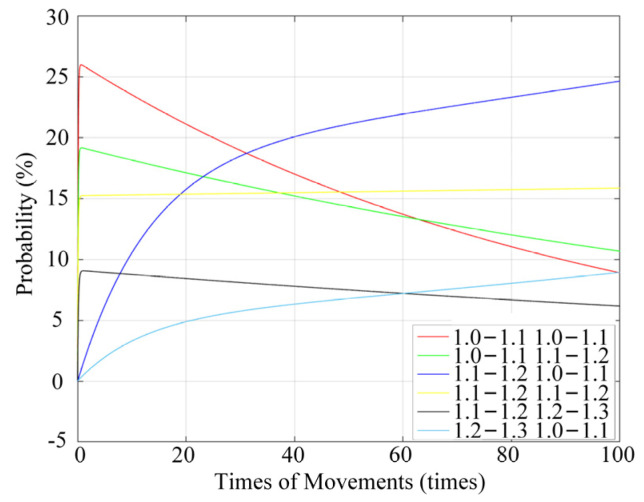
The occurrence probability of an arc center with NO contact changes for different numbers of cycles.

According to the table data, the fitting function is obtained using the second-order exponential method. The occurrence rule of the arc center was analyzed with the fitting function, and the fitting curve is shown in Figure 12.

According to Figure 12, the arc center is offset in the continuous cycles of the relay. In the regions of (1.0–1.1, 1.0–1.1), (1.0–1.1, 1.1–1.2), and (1.1–1.2, 1.2–1.3), the probability of occurrence gradually decreases with the increase in the number of cycles. The probability of occurrence in the region of (1.1–1.2, 1.1–1.2) is relatively stable and is maintained within a certain range. In the regions of (1.1–1.2, 1.0–1.1) and (1.2–1.3, 1.0–1.1), with the increase in the number of cycles, the probability of occurrence gradually increases. Compared to the static contact points, the distribution region of the arc center exhibits slight differences. However, the overall trend in the probability of the appearance of an arc center is similar to that of the static contact points, with arc ablation energy becoming increasingly concentrated. The effect of the ablation degree of the arc on the contact surface is not linear, but with an increase in the number of cycles, the ablation becomes increasingly pronounced. The impact of the arc on surface roughness is initially small and then gradually increases, eventually stabilizing.

Based on the actual measured average roughness of the contact surface, taking a 2-times load as an example, the average roughness of the contact surface changes by 0.0965 when the contact is actuated 20 to 40 times, by 0.2598 when actuated 40 to 60 times, by 0.1918 when actuated 60 to 80 times, and by 0.1918 when actuated 80 to 100 times, which is consistent with the above pattern. Under 4-times and 6-times loads, the average roughness change of the contact surface also follows the trend.

## 3. Establishment of the Equivalent Heat Source Model Based on Surface Roughness

### 3.1. Measurement of Contact Surface Roughness

The contact surface is not a smooth plane but a rough surface with irregularities. When in contact with the movable reed, the actual contact region between the contact and the reed is significantly smaller than the ideal contact region. Therefore, the contact resistance does not remain unchanged as expected but varies with the roughness, leading to changes in the contact temperature rise. To investigate the variations in the actual contact temperature rise, the contact surface roughness was measured, and the true morphology of the contact was objectively reflected through the average surface roughness.

The surface roughness of relay contacts was scanned using a laser confocal scanner under 2-, 4-, and 6-times load with 20, 40, 60, 80, and 100 cycles of operation. The surface roughness of relay contacts without any cycles of operation was also photographed.

**Figure 13:**
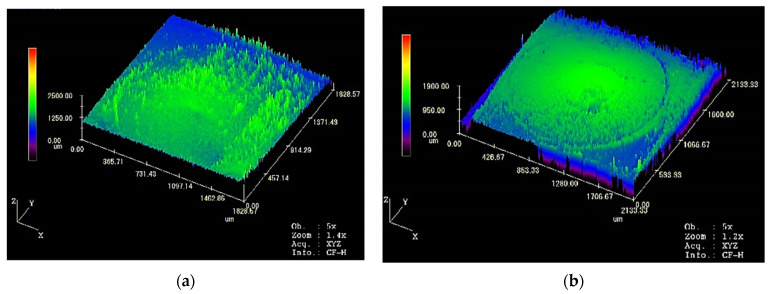
Three-dimensional scanning panorama of contact morphology: (**a**) is the contact morphology of the relay contact without any cycles of operation; (**b**) is the contact morphology after 100 cycles under 6-times load.

A three-dimensional scanning image of the overall morphology of the 6-times load contact is shown in Figure 13.

According to Figure 13, compared to the contact without ablation, a distinct layer is formed on the contact surface after arc ablation. After multiple cycles, ablation marks are formed on the contact surface. The surface roughness undergoes significant change. The impact of the arc on the contact surface is objectively reflected by scanning the average roughness of the contact surface.

**Figure 14:**
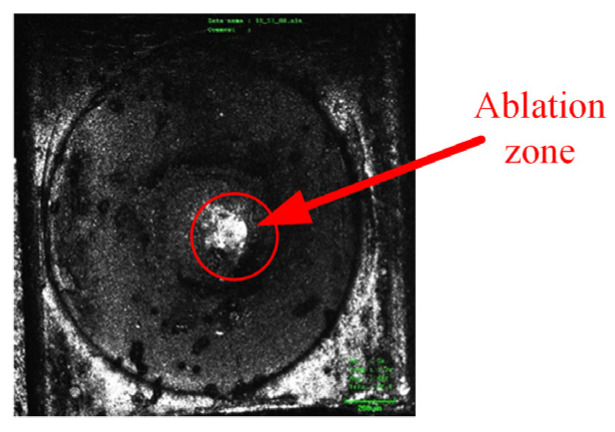
The complete image of the contact after 100 cycles under 6-times load.

The complete image of the contact after 100 cycles under 6-times load is shown in Figure 14.

**Figure 15:**
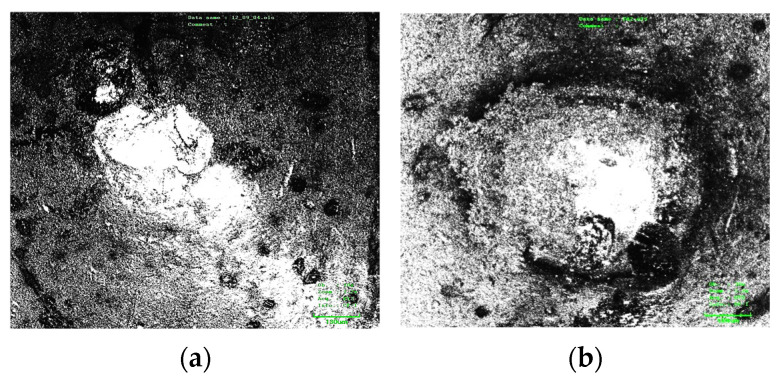
Surface morphology of the ablation region magnified 10 times: (**a**) is the surface morphology without cycles; (**b**) is the surface morphology after 100 cycles under 6-times load.

The surface region of the contact after 100 cycles and without cycles under 6-times load was magnified by 10 times. The specific condition of the contact surface with the movable reed is shown more clearly in Figure 15.

As shown in Figure 15, the surface morphology is relatively smooth and flat, although there are some oxidized spots on the contact without cycles. Under 6-times load, a layer of melted marks is seen on the contact surface after arc erosion, the surface of which is uneven.

**Figure 16:**
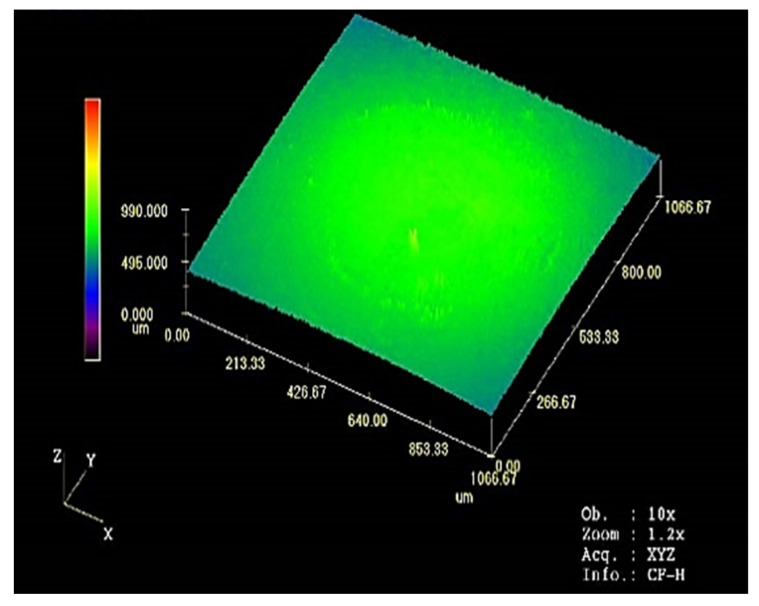
Surface roughness scanning of the contact ablation region for 100 cycles of a 6-times load cycle.

The precision of the average surface roughness data was improved by conducting a more detailed scan by amplifying the surface roughness of the ablation region, as shown in Figure 16.

### 3.2. Measurement of the Contact Surface Roughness

Based on the surface roughness data, the variation in roughness aligns with the analysis from the previous section. As the number of cycles increases, the rate of roughness change gradually accelerates. However, once the roughness achieves a certain threshold, the rate of change stabilizes. Additionally, when the roughness attains a certain level, the rate of change slows down compared to previous intervals of fixed action numbers. The internal material of the contact is eroded by the arc due to the complete ablation of the contact surface coating. Since the internal materials have more stable physical properties, they are relatively consistently and less severely affected by arc erosion. Therefore, for varying levels of load, the higher the load level, the greater the roughness. Below a certain level, the change in roughness is greater, and the number of cycles required to reach the roughness threshold is reduced.

**Figure 17:**
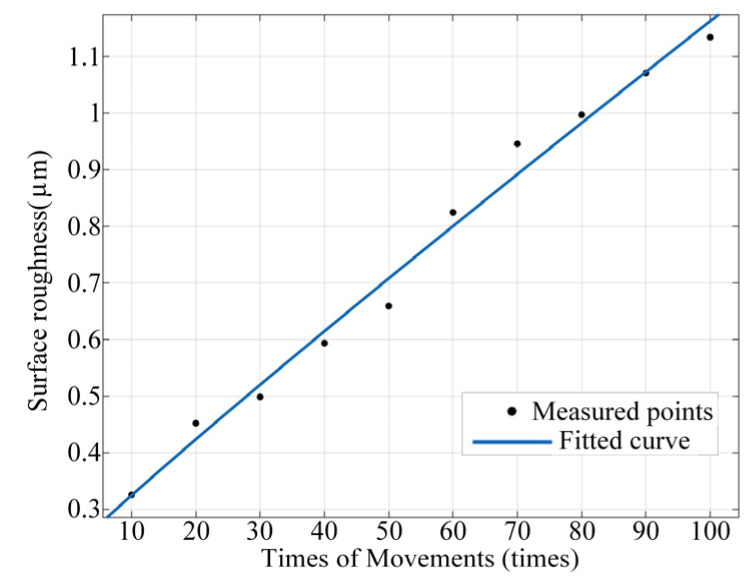
Function of surface roughness variation with the number of cycles under 6-times load.

Taking 6-times load as an example, the surface roughness data and the number of cycles were fitted, as shown in Figure 17.

Due to the minimal error in fitting with a second-order power function, the roughness variation function at 6-times load and 100 cycles is as follows.

(1) $$R_{a}=0.0121x^{0.9465}+0.2178$$

The change rule of the relay contact resistance within 100 actions was predicted according to the changing trend in surface roughness under different load levels within 100 cycles, which is based on the above model. After 100 relay cycles, the maximum surface roughness is 1.1339, and the upper limit of subsequent changes in surface roughness is uncertain. Therefore, the model demonstrated high accuracy only within 100 cycles.

### 3.3. Establishment of Contact Cycles–Contact Resistance Model

The factors affecting the contact resistance of the relay contact surface are the contact pressure, contact material, and surface roughness. As mentioned above, the contact between the contact and the movable reed is not regarded as a contact between smooth planes. At the micro-level, the contact surfaces are regarded as bulges of different heights. When the contact point makes contact with the movable reed, the higher micro-convex body is the first area to make contact. Under a certain contact pressure, the deformation of micro-protrusions on the contact surface is limited, and the movable reed contacts with micro-protrusions within a certain range of heights comprise the actual contact region. Therefore, the contact region is influenced by the contact surface morphology. In fact, as the number of contact cycles increases, the contact surface roughness changes due to the erosion by the arc. Correspondingly, the surface morphology and the height difference of the surface microstructure are altered, leading to the change in the actual contact region during contact.

The surface state of the contact is objectively reflected by the average roughness Ra of the contact surface, which represents the normal average value from each point on the rough surface contour to the centerline. According to ISO 4287 [33], the formula is defined as follows:

(2) $$R_{a}=\frac{1}{l}\int_{0}^{l}\left|z(x)\right|dx$$

Based on the contact cycle frequency–surface roughness model, a contact cycles–contact resistance model was derived from the relationship between contact resistance and surface roughness.

The general definition of contact resistance is as shown in Equation (3) [34]:

(3) $$R_{j}=\frac{\rho }{2a}$$

where *ρ* represents resistivity, and *a* represents contact radius.

In Equation (3), the conductive spot is regarded as the sum of micro-indentations on the contact surface in contact with the movable reed. However, the radius of the conductive spot is generally difficult to measure directly.

Based on the measured average surface roughness, assuming the radius of the contacting ball remains constant and the contact pressure is nearly unchanged, the contact resistance of the contact surface is calculated using the empirical Equation (4):

(4) $$R_{j}=\frac{0.451\left(\theta {R_{a}}^{\beta }\right)}{G\sqrt[{3}]{\frac{Fr_{0}}{E}}}$$

where *G* represents the conductivity of the contact material, *R*_a_ represents the average roughness of the contact surface, *E* represents the elastic modulus of the contact material, *F* represents the contact pressure between the movable reed and the contact, *r*_0_ represents the contact sphere radius between the movable reed and the contact, and *θ* and *β* represent the correction factor.

Assuming the contact pressure is constant, the radius of the contacting sphere is approximately unchanged. The formula for a certain specific contact material with a fixed conductivity and elastic modulus is approximated as a power function:

(5) $$R_{j}=k{R_{a}}^{\alpha }$$

where *k* and *α* represent constants. *k* is measured experimentally or via calculation based on parameters such as the relay contact material and contact pressure. For the HSER in this paper, *k* is approximately 43.4, and *α* = 0.51. *R*_a_ is regarded as a function of the average surface roughness and the number of cycles. Therefore, for different loads, the functional relationship between the contact resistance of the relay and the number of cycles within 100 cycles is as follows.

For the 2-times load,

(6) $$R_{j}=k{\left(0.0067x^{1.0533}+0.1039\right)}^{\alpha }$$

For the 4-times load,

(7) $$R_{j}=k{\left(0.0159x^{0.8935}+0.0934\right)}^{\alpha }$$

For the 6-times load,

(8) $$R_{j}=k{\left(0.0121x^{0.9465}+0.2178\right)}^{\alpha }$$

The following image was drawn according to the data calculated with the above model.

**Figure 18:**
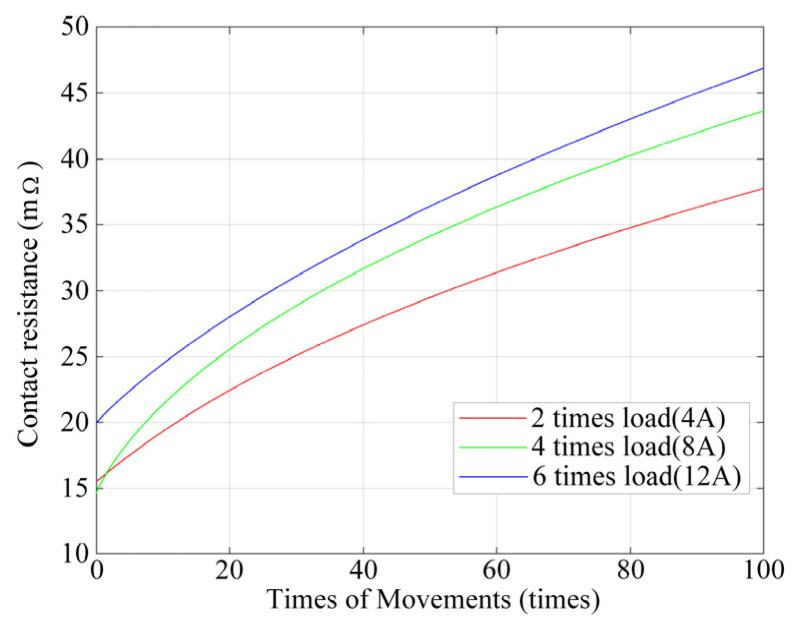
Contact resistance for different loads and numbers of cycles.

According to Figure 18, with an increase in the number of cycles, the rise in speed of contact resistance first tends to be faster and is then slower and tends to be stable. The trend in contact resistance is similar to that of the contact surface roughness, which conforms to the change law between contact resistance and roughness. Under different load conditions, the contact resistance is increased with an increase in load level, which is in line with the microscopic mechanism of electrical contact changes. However, in the case of low numbers of cycles, the calculated contact resistance results exhibit differences from the microscopic mechanism.

The contact resistance calculated with the model was compared with the measured contact resistance data, as shown in Table 3.

According to Table 3, when the number of cycles is between 20 and 100, the contact resistance calculated with the theoretical model is close to that of the experimental results, with an error of less than 10%. However, a contact resistance between 0 and 20 times increases the likelihood of error, which may be caused by the oxide film on the contact surface without any cycles of operation or by the metal coating on the contact surface. After the contact action, the surface oxide film melts, and the metal coating is completely eroded, resulting in significant differences in the contact resistance calculation at a low number of cycles. Due to the small change in contact resistance at lower numbers of cycles, a contact resistance of 20 times was used as a reference.

## 4. Establishment of Equivalent Heat Source Model and Finite Element Simulation of Contact Temperature Rise

**Figure 19:**
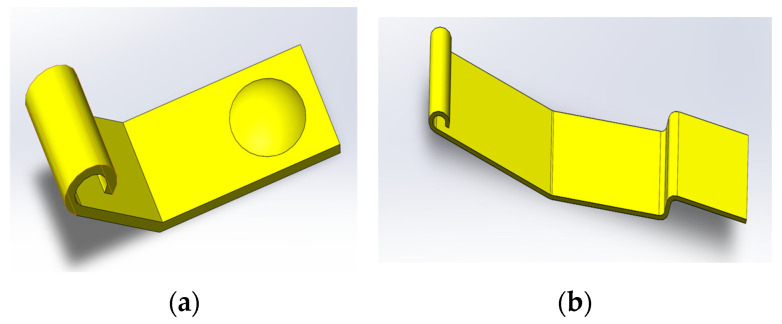
CAD model of contact and movable reed: (**a**) is the CAD model of the contact; (**b**) is the CAD model of the movable reed.

The contact resistance for different loads and different numbers of cycles is calculated with the theoretical model of contact cycles–contact resistance. The temperature rise of the relay contact is derived based on the contact resistance and load current. The contact resistance is substituted into the finite element model to calculate the contact temperature rise of the contact system under different loads. The simulation model is a dynamic spring contact system, and the CAD model is shown in Figure 19.

### 4.1. Establishment of Equivalent Heat Source Model

The equivalent heat source model was established based on the contact action frequency–contact resistance model, and the principle is as follows:

Assuming that the heat source of the HSER is mainly Joule heating, which, based on the Joule thermal law of resistance, is generated by the contact resistance *R*_j_ of the HSER during the operating time, Δ*t* is calculated for a certain current *I*, as shown in Equation (9).

(9) $$Q=I^{2}R_{j}\Delta t$$

Assuming that the heat is hardly dissipated due to the fast action of the HSER, as the number of cycles of the HSER increases, the total heat is the sum of the heat generated by the contact resistance *R*_jn_ within the operating time Δ*t*_n_ of each action. For *k* cycles, the total heat generated by the contact system is shown in Equation (10):

(10) $$Q=\sum_{n=0}^{k}I^{2}R_{\mathrm{jn}}\Delta t_{n}$$

A relationship is established between temperature *T* and contact resistance *R*_j_, based on the heat transfer formula:

(11) Q=CMΔT

(12) $$\sum_{n=0}^{k}I^{2}R_{\mathrm{jn}}\Delta t_{n}=CM\Delta T$$

Under the conditions of a constant load current, contact time, and initial temperature, based on the variation law of contact resistance *R*_jn_, Equation (12) is simplified as (13):

(13) $$I^{2}\bar{R_{\mathrm{jk}}}t=CM\Delta T$$

where $\bar{R_{\mathrm{jk}}}$ represents the average of the contact resistance *R*_j_ within *k* cycles, *t* represents the total contact time within *k* cycles, and *I* represents the load current. The specific heat capacity *C* is a constant for the same contact.

Assuming the mass *M* of the HSER constant and the initial temperature is *T*_c_, the heat source temperature *T* is calculated with the following formula:

(14) $$\Delta T=\frac{\bar{R_{\mathrm{jk}}}\cdot t\cdot I^{2}}{CM}$$

(15) $$T=\Delta T+T_{c}$$

Based on Equation (15) and the contact cycles–contact resistance model, the heat source temperature of the HSER is calculated for different numbers of cycles under 2-, 4-, and 6-times load. The temperature distribution between the movable reed and the contact was simulated.

### 4.2. Establishment and Analysis of Contact Temperature Rise Simulation Model

**Figure 20:**
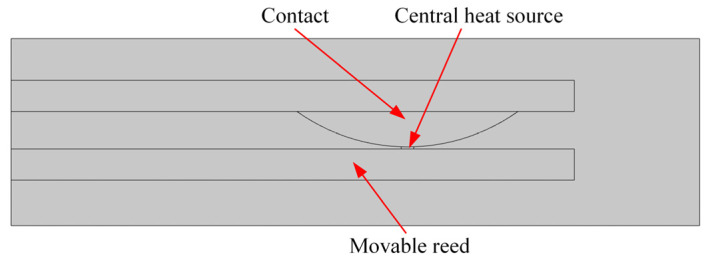
Dynamic reed–contact simulation model.

**Figure 21:**
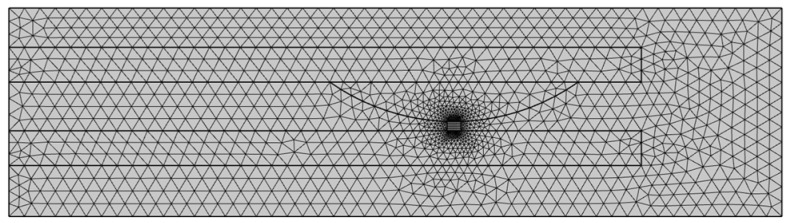
Dynamic reed–contact meshing model.

The model of the movable reed and contact was established. This model involves a certain type of HSER, as shown in Figure 20. The static contact model was meshed, as shown in Figure 21.

The boundary conditions of the model are as follows, 4 A 28 V DC, 8 A 28 V DC, and 12 A 28 V DC. The equivalent heat source is loaded on the contact between the contact and the movable reed, and the contact temperature rise is calculated for different numbers of cycles.

The temperature cloud map of the movable reed and contact and the contact temperature rise is calculated.

**Figure 22:**
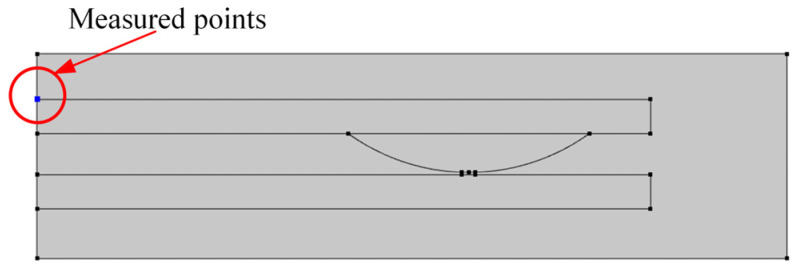
Schematic diagram of the measured point.

Selection of the endpoint of the contact to measure the temperature rise is considered an approximation of the contact pin temperature to compare it with the experimental temperature rise, as shown in Figure 22.

**Figure 23:**
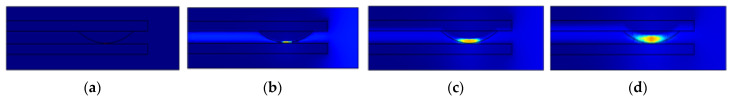
Simulated temperature cloud map: (**a**) is the temperature cloud map at 0 s (the beginning of the heat transfer); (**b**) is the temperature cloud map at 0.5 s (during the heat transfer); (**c**) is the temperature cloud map at 1.0 s (during the heat transfer); (**d**) is the temperature cloud map at 2.0 s (the end of the heat transfer).

A temperature cloud map was generated by simulating the temperature rise of each part. The temperature rise cloud diagram of an HSER with 20 cycles under 6-times load is taken as an example, as shown in Figure 23.

**Figure 24:**
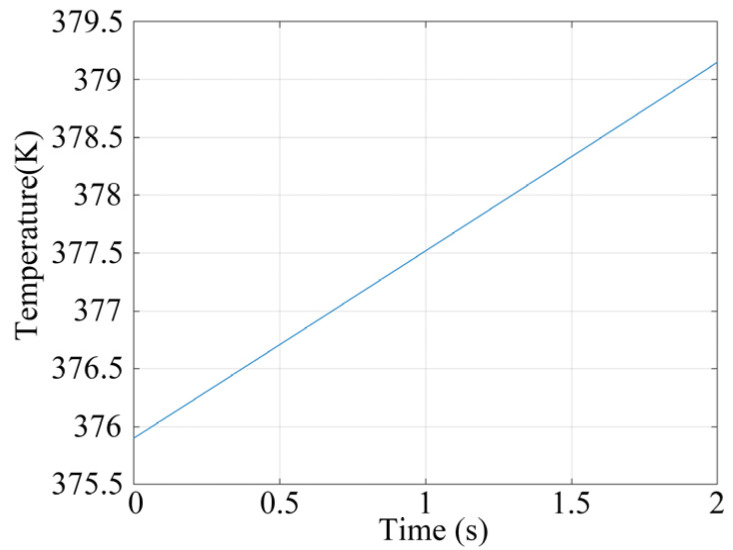
Temperature curve of the measured point.

From Figure 24, it can be observed that the final temperature at the measured point is 379.17 K (106.02 °C) of 20 cycles under 6-times load.

Following the above process, the temperature rise of the measured point for 20, 40, 60, 80, and 100 cycles of the HSER under 2-, 4-, and 6-times load was calculated.

## 5. Experiment and Analysis of Temperature Rise in Contact System

### 5.1. Composition and Establishment of Thermal Characteristic Testing System

**Figure 25:**
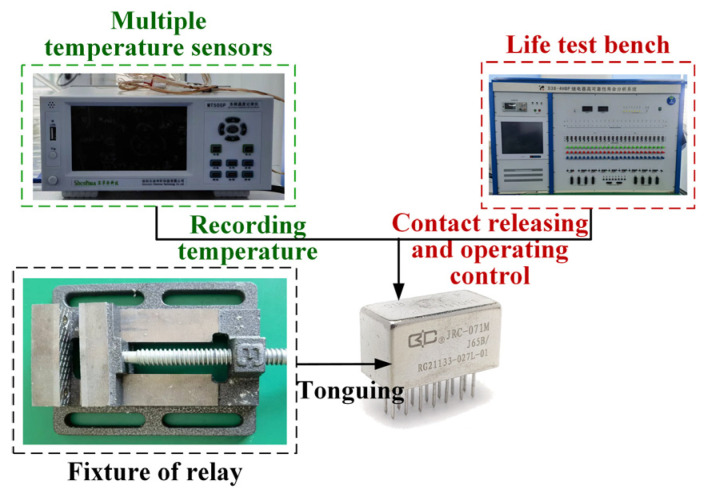
The system structure of the thermal characteristic testing system.

The thermal characteristic testing system is mainly composed of a high-reliability relay life analysis system and a multi-channel temperature recorder. The overall system structure of the thermal characteristic testing system is shown in Figure 25.

The main measurement contents are as follows: a temperature rise curve of 10,000 cycles under rated load for analyzing the temperature change of the relay and a temperature rise curve of 100 cycles under 2-, 4-, and 6-times load to verify the accuracy of the contact cycle–contact resistance model.

The pin of the movable reed in the NO contact set, the pin of the steady reed in the NO contact set, the pin of the movable reed, and the pin of the positive pole of the coil were used to connect the thermocouples to the case, and the temperature rise of the HSER was measured.

### 5.2. Temperature Rise Experiments of HSER Under Rated Load

Firstly, the temperature rise of a certain model of an HSER under rated load was measured, and the temperature rise changes of the HSER were analyzed. The law of change is summarized. The environment temperature was 25.4 °C.

**Figure 26:**
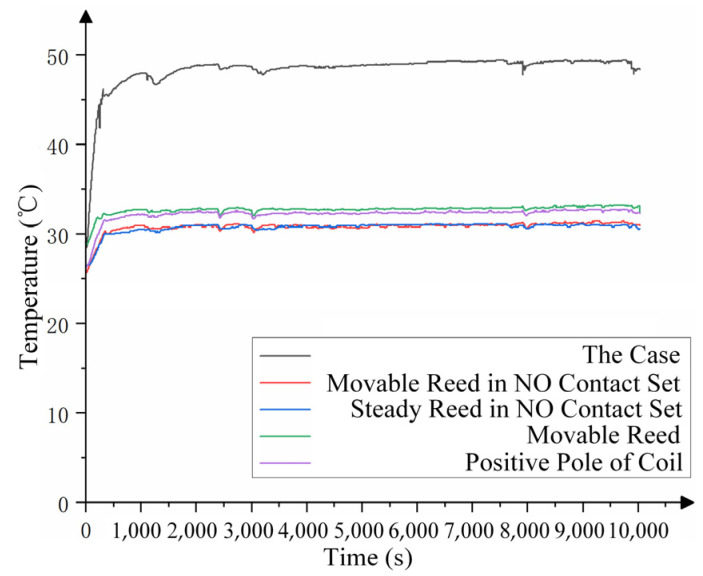
Temperature curve for 10,000 cycles under rated load (2 A).

The load was 28 V/2 A, the coil voltage was 28 V/1 A, the number of cycles was 10,000, and the total time was 10,000 s. The temperature of each part is shown in Figure 26.

As shown in Figure 26, the temperature of each part is increased first, and after a certain period, the temperature tends to stabilize. When cycles continue after reaching the stable temperature, the temperature of each part fluctuates slightly with the stable temperature, and an overall steady state is attained.

### 5.3. HSER Experiments Under Overload Conditions

The temperature rise characteristics of the HSER are summarized by measuring the temperature rise under rated load. To verify the accuracy of the theoretical model, the transient temperature was measured after 100 cycles under 2-, 4-, and 6-times loads. The environment temperature was 25.4 °C.

**Figure 27:**
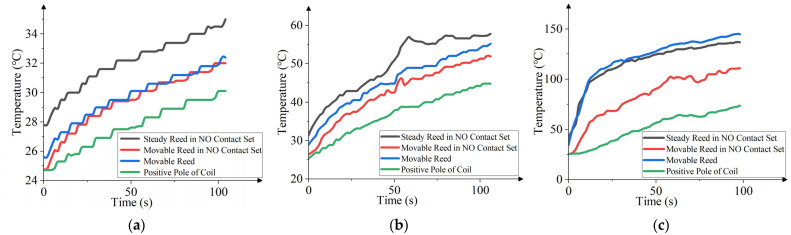
Transient temperature curve for 100 cycles under different loads: (**a**) is the transient temperature curve under 2-times load (4 A); (**b**) is the transient temperature curve under 4-times load (8 A); (**c**) is the transient temperature curve under 6-times load (12 A).

The loads were 28 V/4 A, 28 V/8 A, and 28 V/12 A; the number of cycles was 100; and the total time was 100 s. The temperature of each part is shown in Figure 27.

The transient temperature of the HSER is a zigzag upward trend with an increase in relay action time. The greater the load, the faster the temperature rises. Under 2-times load, each part of the relay reaches a steady state. Finally, the temperature of the steady reed in the NO contact set is the highest, and the temperature of the movable reed and the movable reed in the NO contact set are similar, both higher than the temperature of the positive pole of the coil. Under 4-times load, the temperature trend of each part is similar to that of 2-times load, but due to the increase in the load, the steady reed in the NO contact set quickly reaches a steady state. Under 6-times load, the steady reed in the NO contact set and movable reed reach a steady state first, followed by the movable reed in the NO contact set. The positive pole of the coil does not achieve a steady state. The temperature of the movable reed and steady reed in the NO contact set is increased the most, the rising speed of which is the fastest. Assuming that the load is too large, the heat dissipation of the movable reed is far lower than that of the current under the influence of a large current. With the same number of cycles, the temperature of each part under 6-times load is significantly higher than that under 4-times load.

### 5.4. Experimental Verification of Equivalent Heat Source Model

The equivalent heat source model based on surface roughness was substituted into the finite element simulation model, and the temperature rise of the contact pin was calculated. This provided data support for the validation of the equivalent heat source model.

The contact temperature rise according to the theoretical equivalent heat source model above was compared with the experimental results, as shown in Table 4.

According to Table 4, the error between the contact temperature rise according to the equivalent heat source simulation and that of the experimental results is less than 10%. The equivalent heat source model is considered a more accurate description of the change law of the contact temperature rise with the number of relay cycles.

## 6. Discussion

In this paper, the arc center trajectory and the arc ablation region distribution were collected via the experimental method firstly. The movement law of the arc was summarized. The influence of the arc on the surface roughness of the contact was analyzed. Based on the collected surface roughness data and the relationship between the contact resistance and the surface roughness, the equivalent heat source model of thermal relay contact based on the surface roughness of silver–magnesium–nickel contact was established, and the accuracy of the model was verified through the experiment.

The equivalent heat source model of thermal relay contact based on the surface roughness of silver–magnesium–nickel contact in this paper can be used for calculating the contact temperature rise of silver-based contact relays under high overload conditions. Compared to the traditional calculation of the contact resistance, the real situation of the contact surface was more accurately simulated via the theoretical model in this paper through introducing surface roughness variables, making the calculation of the contact resistance more realistic.

Under different loads for the mathematical model of the contact resistance in this paper, the contact resistance increases with the increase in load level, which conforms to the microscopic mechanism of electrical contact changes. However, in the case of low action cycles, the calculated results of the contact resistance were different from the microscopic mechanism.

The research in this paper was limited to experimental conditions, such as the resolution of high-speed cameras and scanning electron microscopes of roughness and methods of image processing. The frame rate of clear arc images under the same arcing time was affected by the resolution of high-speed cameras. More arc captures were collected via higher resolution high-speed cameras. The accuracy of the contact surface roughness data and the precision of surface morphology were affected by the resolution of roughness scanning electron microscopy. More accurate surface roughness data and more realistic surface morphology were collected via higher resolution scanning electron microscopy. In this paper, the arc trajectory and ablation area on the contact surface were extracted via the contour extraction of the image processing software. On this basis, the step of image preprocessing can be added, such as gray transformation enhancement. The arc trajectory and the ablation region were more accurate through image segmentation methods such as region growing algorithms. In the future, the above aspects can be improved to further optimize the model.

Based on the research in this paper, the model was expanded into other mathematical models and optimized to be more universal by changing the experimental conditions. For example, the experimental ambient temperature in this paper is 25.4 °C. By increasing or decreasing the experimental ambient temperature, the micro mechanism was analyzed to establish new mathematical models for different ambient temperatures or the expanded mathematical model combining the experimental data in this paper. Similarly, the changed factors were the experimental atmosphere, the breaking speed, the contact pressure, the contact material, etc. The research object of this paper was the contact between spheres and planes. Other contact shapes such as plane to plane, sphere to sphere, or spheres with different radii can be researched under the same experimental conditions.

## 7. Conclusions

The arc center trajectory and arc ablation region distribution were drawn by building a two-dimensional synchronous double-axis arc photographing platform. Compared with the experimental results, the error is less than 10%. The movement law of the arc was determined by calculating the probability of an arc center appearing in different positions. The nonlinear characteristics of the influence of the arc on the contact surface roughness was predicted.

A mathematical model of the contact cycles–surface roughness was established. On this basis, a mathematical contact cycles–contact resistance model was established through the relationship between the contact resistance and surface roughness. According to thermodynamic equations, the equivalent heat source model for thermal relay contact based on contact surface roughness was established. By substituting the model into the FEM model, the contact temperature rise of an HSER with different loads and numbers of cycles was calculated, and the theoretical model error was less than 10%.

Based on the thermal characteristic testing system, the temperature rise of the relay contact under rated load and overload conditions was measured, and the trend in the relay working temperature change was summarized. The accuracy of the equivalent heat source model was verified by comparing the experimental data with those of the simulation.

In future work, the arc trajectory can be upgraded from two-dimensional to three-dimensional, further accurately summarizing the arc movement law. The optimization of relay structure design and the prediction of relay lifespan are of greater significance.

## Figures and Tables

**Table 1:** Position of NO contact arc on coordinate axis.

Frame Number	Left End of *X* Axis (mm)	Right End of *X* Axis (mm)	Center of *X* Axis (mm)	Left End of *Y* Axis (mm)	Right End of *Y* Axis (mm)	Center of *Y* Axis (mm)
1	0.990	1.260	1.125	0.968	1.232	1.100
2	0.945	1.260	1.103	0.939	1.203	1.071
3	0.900	1.215	1.058	0.968	1.173	1.071
4	0.855	1.215	1.035	1.027	1.261	1.144
5	0.900	1.260	1.080	0.968	1.203	1.086
6	0.765	1.215	0.990	0.909	1.261	1.085
7	0.810	1.350	1.080	0.997	1.232	1.115
8	0.855	1.350	1.103	0.997	1.320	1.159
9	0.900	1.305	1.103	0.821	1.232	1.027
10	0.765	1.305	1.035	0.909	1.203	1.056
11	0.945	1.305	1.125	1.056	1.291	1.174
12	0.900	1.350	1.125	1.027	1.261	1.144
13	0.945	1.350	1.148	1.027	1.232	1.130
14	0.810	1.305	1.058	0.880	1.203	1.042
15	0.945	1.260	1.103	0.909	1.203	1.056

**Table 2:** Occurrence probabilities of an arc center in the region.

*X*-Axis (mm)	*Y*-Axis (mm)	20 Times (%)	40 Times (%)	60 Times (%)	80 Times (%)	100 Times (%)
1.0–1.1	1.0–1.1	22.22	16.38	11.65	11.09	10.75
1.0–1.1	1.1–1.2	17.95	14.22	13.35	12.15	11.09
1.1–1.2	1.0–1.1	15.38	21.12	21.59	22.39	25.26
1.1–1.2	1.1–1.2	16.24	14.22	15.34	17.06	15.36
1.1–1.2	1.2–1.3	8.55	7.76	7.10	6.61	6.31
1.2–1.3	1.0–1.1	5.13	5.60	7.95	7.89	8.87
1.2–1.3	1.1–1.2	3.42	4.31	4.26	4.90	4.95

**Table 3:** Comparison of contact resistance between theoretical model and experimental results for different loads and numbers of cycles.

Number of Cycles	2-Times Load			4-Times Load			6-Times Load		
	Theoretical Model (mΩ)	Experiment (mΩ)	Error (%)	Theoretical Model (mΩ)	Experiment (mΩ)	Error (%)	Theoretical Model (mΩ)	Experiment (mΩ)	Error (%)
0	15.52	20.55	−24.48	14.59	21.06	−30.72	19.95	20.49	−2.64
20	22.44	22.24	0.90	25.55	24.55	4.07	28.01	27.11	3.32
40	27.40	25.37	8.00	31.67	29.82	6.20	33.87	34.76	−2.56
60	31.39	29.16	7.65	36.35	36.47	−0.33	38.76	41.5	−6.60
80	34.78	32.82	5.97	40.26	41.65	−3.34	43.03	45.45	−5.32

**Table 4:** Comparison of contact temperature rise between theoretical model and experimental results for different loads and numbers of cycles.

Number of Cycles	2-Times Load			4-Times Load			6-Times Load		
	Theoretical Model (°C)	Experiment (°C)	Error (%)	Theoretical Model (°C)	Experiment (°C)	Error (%)	Theoretical Model (°C)	Experiment (°C)	Error (%)
20	28.6	30.1	−4.98	39.5	41.8	−5.5	106.0	103.8	2.12
40	29.4	31.6	−6.96	42.8	45.8	−6.55	125.4	117.9	6.36
60	31.5	32.8	−3.96	56.2	56.9	−1.23	135.8	127.4	6.59
80	33.5	33.4	0.30	61.6	57.2	7.69	141.9	132.1	7.42
100	36.8	34.5	6.67	62.8	57.8	8.65	143.4	135.7	5.67

## Data Availability

The original contributions presented in this study are included in this article. Further inquiries can be directed to the corresponding author.

## References

[1] Yang W.Y., Xie M., Chen Y.T., Wang J.H., Zhang J.M., Wang S. (2016). Microstructure and Phase Analysis of Ag-Mg-Ni Alloy. Chin. J. Rare Met..

[2] Wang Z.H. (2007). Analysis on Reliability and Contact Bonding for Sealed Electromagnetic Relay. Master Thesis.

[3] Shi H.W., Zhang G.Y., Cheng Z.M., Lu Y., Xiong Y.N. (2023). Common Failure Types Analysis on Automobile Relay. Auto Electr. Parts.

[4] You J.X., Fu R., Liang H.M., Zhang J.H., Feng X.D. Failure Simulation and Analysis Method of Hermetically Sealed Eletromagnetic Relay Under Long-Term Load. Proceedings of the 2020 IEEE 66th Holm Conference on Electrical Contacts and Intensive Course (HLM).

[5] Li X.F. (2004). Simulation of Low Voltage Apparatus Switching Arc. Ph.D. Thesis.

[6] Gordon L.B. Modeling DC Arc Physics and Applications for DC Arc Flash Risk Assessment. Proceedings of the 2023 IEEE IAS Electrical Safety Workshop (ESW).

[7] Li Y.Z., Wang Z.H., Li Q.M. Vacuum Arc Characteristics of a Novel Contact Consists of Arcing Contact and Main Contact. Proceedings of the 2019 5th International Conference on Electric Power Equipment-Switching Technology (ICEPE-ST).

[8] Ren L.L., Wang Z.B., Wang S., Li C.D., Wang W., Ming Z., Zhai Y.C. (2023). The Effect of Cu Content on The Microstructure and Properties of The Wire Arc Additive Manufacturing Al-Cu Alloy. Mater. Sci..

[9] Samal S., Blanco I. (2022). An Overview of Thermal Plasma Arc Systems for Treatment of Various Wastes in Recovery of Metals. Mater. Sci..

[10] Lu S., Sun X.W., Zhang B.W., Wu J.S. (2024). An Review of Cathode Plasma Electrolysis Treatment: Progress, Applications, and Advancements in Metal Coating Preparation. Mater. Sci..

[11] Jin-Ho P., Sang-Youl K., Han-Seung L., Kwangwoo W. (2023). Influence of Different Metal Types on the Bonding Strength of Concrete Using the Arc Thermal Metal Spraying Method. Mater. Sci..

[12] Sawa K., Yoshida K., Watanabe M., Suzuki K. Arc Characteristics and Electrode Mass Change of AgNi Contacts for Electromagnetic Contactors. Proceedings of the 2010 56th IEEE Holm Conference on Electrical Contacts (HLM).

[13] Zhou X., Zhou Y.X., Sun H.C., Zhai G.F. (2021). Study on Arc Characteristics in a Self-Blowing Arc Chamber for a DC High Power Contactor. Trans. China Electrotech. Soc..

[14] Wang H.T., Yang B., Zheng S.Q., Liang Y.X. (2024). Simulation Study on Arc Dynamic Characteristics of DC Contactor Under Metallic Grid Sheet. Transducer Microsyst. Technol..

[15] Bo X.L., Liu S.Y., Xie H.T., Wang X.J., Feng W.G., Li P., Liu Z.Y. (2024). Vacuum Arc Ignition and Ablation Characteristics of CuCr55 Electrode Contacts. High Volt. Appar..

[16] Hu X.F., Gao H.Y., Mao J.H. (2004). Discussing the Effects of Contact Surface Coarseness on Contact Resistance. Electr. Eng. Mater..

[17] Deac C.N., Adam M., Andrusca M., Dragomir A. Aspects Regarding Contact Resistance Measurement. Proceedings of the 2019 8th International Conference on Modern Power Systems (MPS).

[18] Bai X.P., Li G.W., Weng W., Zhang M.J., Lin W.H., Zhu L.B., Zhang Y.P. (2013). Effects of Contact Surface Condition on Contact Resistance and Improving Methods. Electr. Eng. Mater..

[19] Jedliński M., Krupa J., Janiszewska-Olszowska J. (2024). The Micromechanical Properties and Surface Roughness of Orthodontic Retainer Wires—An In Vitro Analysis. Mater. Sci..

[20] Wang K., Gao Y.F., Yang C.F. (2024). Prediction of Subsurface Microcrack Damage Depth Based on Surface Roughness in Diamond Wire Sawing of Monocrystalline Silicon. Mater. Sci..

[21] Zagórski I., Szczepaniak A., Kulisz M., Korpysa J. (2022). Influence of the Tool Cutting Edge Helix Angle on the Surface Roughness after Finish Milling of Magnesium Alloys. Mater. Sci..

[22] Maier R., Bucaciuc S., Mandoc A.C. (2022). Reducing Surface Roughness of 3D Printed Short-Carbon Fiber Reinforced Composites. Mater. Sci..

[23] Wang D.Y. (2021). Surface Roughness Characteristics and Contact Resistance Mathematical Model of Sliding Electrical Contact. Master Thesis.

[24] Lu W.J. (2021). Research on The Influence of Microscopic Characteristics of Contacts on The Contact Performance of Railway Relay. Master Thesis.

[25] Guan X., Li W.H. (2018). Analysis of 3D Morphology Characteristics and Contact Performance of Electromagnetic Relay Contacts. Bull. Sci. Technol..

[26] Li R.Y., Yang W.Y., Liang H.M. (2020). Multi-Physics Finite Element Model of Relay Contact Resistance and Temperature Rise Considering Multi-Scale and 3D Fractal Surface. IEEE Access.

[27] Pieszka-Łysoń M., Rutkowski P., Kawalec M., Kawalec D. (2024). Determination of Contact Resistance of Thermal Interface Materials Used in Thermal Monitoring Systems of Electric Vehicle Charging Inlets. Mater. Sci..

[28] Kondo A., Matsuura H., Ito Y. (2023). Numerical Study on Effect of Contact and Interfacial Resistance on Thermal Conductivity of Dispersed Composites. Mater. Sci..

[29] Zhang M.Y., Tan Y.X., Yang C., Deng J., Xie Z.C. (2023). Analysis and Optimization of Contact Material Ablation under the Cumulative Effect of the Number of Breakings of OLTC. Mater. Sci..

[30] Yevtushenko A., Topczewska K., Zamojski P. (2023). The Mutual Influence of Thermal Contact Conductivity and Convective Cooling on the Temperature Field in a Tribosystem with a Functionally Graded Strip. Mater. Sci..

[31] Zheng Y.X., Fan X.M., Zhang W.J., Zhang X. Steady-State Thermal Analysis Simulation of Magnetic Holding Relay Contact Based on Finite Element Analysis. Proceedings of the 2019 12th International Symposium on Computational Intelligence and Design (ISCID).

[32] Zhao Z.E. (2021). Study on Loop Resistance and Temperature Rise of Contact Spring System within Relays. Master Thesis.

[33] (1997). Geometrical Product Specifications (GPS)-Surface Texture: Profile method-Terms, Definitions and Surface Texture Parameters.

[34] Ragnar H. (2010). Electric Contacts.

